# Additional measures of macular function beyond visual acuity

**DOI:** 10.1007/s00417-023-06272-1

**Published:** 2023-11-08

**Authors:** Hernán Andrés Ríos, Monica Lövestam-Adrian, Sotiris Plainis, Miltiadis Tsilimbaris, Antonia M. Joussen, David Keegan, Martin Charles, José Cunha-Vaz, Edoardo Midena

**Affiliations:** 1 Retina y Vítreo, Fundación Oftalmológica Nacional, Universidad del Rosario, Bogotá, Colombia; 2 Department of Ophthalmology, Lund University Hospital, Lund, Sweden; 3 Laboratory of Optics and Vision, University of Crete Medical School, Heraklion, Crete, Greece; 4 Charité – University Medicine Berlin, Berlin, Germany; 5 Department of Ophthalmology, Mater Misericordiae University Hospital, Dublin, Ireland; 6 Charles, Centro Oftalmológico, Buenos Aires, Argentina; 7 AIBILI – Association for Innovation and Biomedical Research on Light and Image, Coimbra, Portugal; 8 Department of Ophthalmology, University of Padova, Padua, Italy; 9 IRCCS Fondazione Bietti, Rome, Italy

**Keywords:** Vision, Visual acuity, Psychophysical test, Electrophysiological test

## Abstract

**Purpose:**

Visual function is a complex process in which external visual stimuli are interpreted. Patients with retinal diseases and prolonged follow-up times may experience changes in their visual function that are not detected by the standard visual acuity measure, as they are a result of other alterations in visual function. With the advancement of different methods to evaluate visual function, additional measurements have become available, and further standardization suggests that some methods may be promising for use in clinical trials or routine clinical practice. The objectives of this article are to review these additional measurements and to provide guidance on their application.

**Methods:**

The Vision Academy’s membership of international retinal disease experts reviewed the literature and developed consensus recommendations for the application of additional measures of visual function in routine clinical practice or clinical trials.

**Results:**

Measures such as low-luminance visual acuity, contrast sensitivity, retinal fixation and microperimetry, and reading performance are measures which can complement visual acuity measurements to provide an assessment of overall visual function, including impact on patients’ quality of life. Measures such as dark adaptation, color vision testing, binocular vision testing, visual recognition testing, and shape discrimination require further optimization and validation before they can be implemented in everyday clinical practice.

**Conclusion:**

Additional measurements of visual function may help identify patients who could benefit from earlier diagnosis, detection of disease progression, and therapeutic intervention. New and additional functional clinical trial endpoints are required to fully understand the early stages of macular disease, its progression, and the response to treatment.


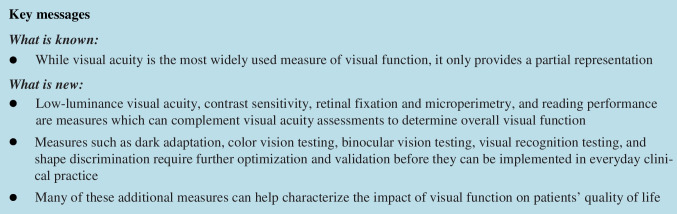


## Introduction

Visual function is a complex process involving multiple interactions between the eye and the brain. These intricate processes are influenced by many factors, including both external environmental factors (e.g., target luminance and contrast, ambient illumination) and internal factors attributed to ocular and brain conditions (e.g., refractive media opacity, retinal lesions, brain cortex damage) [1–3]. Visual acuity (VA) is the most commonly used measure of visual function [4]. By quantifying the minimum visual angle of resolution, VA provides a single measurement of a patient’s visual function [5]. However, vision in daily life depends on varying dimensions, including spatial frequency, spatial location, and contrast [6], so measuring visual function is not straightforward. Some patients with normal VA often report impairment or difficulty with everyday tasks [5, 7], while others may experience an improvement in VA and a reduction in foveal thickness in parallel with other impairments, such as a lack of color vision recovery [8].

Even well-established methods of measuring visual function have numerous inefficiencies. For example, the Amsler grid is a commonly used tool to evaluate a specific parameter of visual function (metamorphopsia) and performance in patients with central retinal impairment, but it lacks reliable reproducibility [9], making it insufficient to quantify and follow-up in most cases. Additionally, VA measurements may not be sensitive enough to detect the slow progression of all components of visual function. Therefore, additional measurements may help identify patients who could benefit from earlier diagnosis, detection of disease progression, and therapeutic intervention [10].

Different methods have facilitated the understanding and measurement of many aspects of visual function, and additional measurements may provide an opportunity to better characterize a patient’s vision and its impact on day-to-day functioning and quality of life. Visual function can be evaluated according to multidimensional factors, and psychophysical and electrophysiological methods can be applied for better evaluation. Tests include low-luminance VA (LLVA), contrast sensitivity (CS), dark adaptation, retinal fixation, color discrimination, reading performance, visual recognition, and shape discrimination. However, some of these tests require high cooperation from the patient. Electrophysiological methods represent a more objective evaluation of visual function and include electroretinography, multifocal electroretinography, and visual evoked potentials [10–14]. However, these latter methods are significantly more time-consuming and will require further standardization before adaptation as routine clinical practice.

The objectives of this article are to review the aforementioned measures of visual function and to provide recommendations on their application to clinical practice and clinical trials. It is not intended as an exhaustive review of all the available measures of visual function but rather as a brief overview of those that are most widely used, with guidance for practicing ophthalmologists on their advantages, limitations, and indications. The article is based on a review of the literature and a consensus among retinal experts who are members of the Vision Academy, an international group of retinal physicians who work together to share existing skills and knowledge and provide collective recommendations on clinical challenges in areas where there is a lack of conclusive evidence in the literature (www.visionacademy.org).

Recommendations were developed by the authors and subsequently reviewed, commented upon, and endorsed by a majority of the Vision Academy membership. Vision Academy members were asked to rate their agreement with the proposed recommendations using the options “strongly agree”, “agree”, “neither agree nor disagree”, “disagree”, and “strongly disagree”. Responses from more than 50% of members were required for the survey to be valid. Respondents were also asked for the reimbursement status of treatment in their country of practice (i.e., mostly reimbursed or mostly out of pocket) to determine if this may have influenced their responses. Biases were assessed using χ^2^. Endorsement of the recommended measures of visual function was established if 50% or more of the respondents indicated that they agreed or strongly agreed. The list of Vision Academy members who have contributed to the recommendations is provided within the “Acknowledgements” section of the article.

## Parameters of VA (best-corrected VA) and visual function

VA is defined as the ability to identify subtle differences in the environment. It is measured according to visual stimuli, with excellent VA indicating that the image is clearly focused on the retina, the visual pathway is functioning correctly, and appropriate interpretation of the visual stimuli has occurred [15]. VA assessment appears to be a simple method of obtaining a fast and reliable measure of a patient’s visual function, as it has minimal cost and risk to the patient, it can be performed quickly and easily, and there is a high prevalence of detectable abnormalities [2, 15]. However, taking into consideration all information acquired through vision, it could be concluded that VA measurements, such as using the Snellen chart, are not sufficient for an integral evaluation of visual function. VA is only one aspect of visual function, intended to quantify the minimum visual angle of resolution. Aspects such as distortion, contrast, dark adaptation, color, and fixation are not evaluated when a Snellen chart test is used but are all important for a comprehensive assessment of visual function [6, 9, 16–20]. Complementary methods to the Snellen chart have recently become a topic of clinical research, as they would be very useful in the clinic for the evaluation of visual function in patients with visual impairment.

## Recommended measures of visual function

VA is the most frequently used tool to measure visual function, although there are many other tests that could be complementary. In this paper, we review and explain the most commonly used tests and highlight recommendations for, and the limitations of, each of the tests to provide key guidance when considering using any of the tests (Table 1). We recommend the use of LLVA, CS, retinal fixation and microperimetry, and reading performance as complementary measures to visual acuity for the assessment of overall visual function.

**Table 1:** Summary of recommendations for measuring visual function

Measure	When and where to use the measure and why	Advantages	Barriers to use in clinical practice and/or clinical trials	Specific recommendations for application
**Recommended measures of visual function**				
Low-luminance visual acuity	• Indications: AMD, DME, central serous chorioretinopathy, PDR (PRP), and IRD • Follow-up for patients with dry AMD	• Simple, inexpensive, and rapid measure	• Should be well explained to the patient to inform them that best-corrected visual acuity will naturally drop	• Using a 2.0-log unit neutral-density filter • Larger benefit in non-neovascular AMD • A self-administered test could be considered
Contrast sensitivity	• Indications: AMD, DME, refractive surgery, central serous chorioretinopathy, PDR (PRP), and IRD • After PRP in patients with diabetes as they often experience visual discomfort with good visual acuity • Evaluation of patients for whom visual acuity does not match their reported visual problems	• Rapid measure • Linked to vision-related quality of life	• Variability of results • Influenced by media opacities, namely cataracts	• Use of a computer-controlled screen is preferable
Retinal fixation and microperimetry	• Indications: AMD, DME, vitreoretinal disorders, retinotoxicity disorders, macular dystrophies, and IRD • Better correlation and understanding of morphology (i.e., imaging) and function, especially retinal sensitivity	• Good correlation between retinal fixation and reading performance	• Equipment not available at all retinal clinics • Long testing duration traditionally but duration has improved with recent developments	• Use short-duration testing strategies • Print out results with probability maps of disease progression • Use to determine fixation in advanced AMD
Reading performance	• Indications: AMD, DME, vitreoretinal disorders, refractive surgery • Follow-up visits to evaluate response after anti-VEGF treatment for AMD or DME • Better assessment of the impact of visual impairment on quality of life than ETDRS charts	• Strongly linked to vision-related quality of life	• Lack of standardization • Lack of agreement on methodology • Depends on a patient’s literacy	• Example: Radner reading charts • Comparability needs to be ensured • May be performed uni- or binocularly
**Measures of visual function requiring further optimization**				
Dark adaptation	• Indications: AMD, DME, PDR (PRP) • To differentiate AMD from variants of genetic disease • Early diagnosis of AMD progression if short-duration testing strategies prove effective	• Assesses photoreceptor dynamic response	• Lack of standardization • Long testing duration (time-consuming) • Requires special examination equipment and conditions which are not always available (i.e., a dedicated dark room)	• Use short-duration testing strategies
Binocular vision testing	• Indications: neurological disorders, squinting, IRD, and nystagmus • Driver’s license testing in some countries • Evaluation of real-life visual performance for medical or legal purposes	• Meaningful for real-life activities • Strongly linked to vision-related quality of life	• Underestimation of monocular visual changes • Lack of standardization	• Not applicable
Color vision testing	• Indications: primarily diabetic retinopathy, DME • Driver’s license testing in some countries • Neurological disorders • IRD	• Easily performed • Standardized (printed charts)	• Influenced by media opacities, namely, cataracts • Tests a different function from visual discrimination, so there is limited correspondence with other tests	• Use of Cambridge Colour Test or other computerized tests is faster than the classic print-based tests (e.g., Ishihara, Farnsworth)
Visual recognition tests	• Indication: AMD • To differentiate from neurological or cognitive disorders such as Charles Bonnet syndrome	• Linked to vision-related quality of life	• Lack of standardization • Limited relevance for monitoring AMD progression	• Not applicable
Shape discrimination	• Indications: AMD, DME • To differentiate from neurological or cognitive disorders such as Charles Bonnet syndrome	• Linked to vision-related quality of life	• Lack of standardization	• Can be used for self-monitoring of AMD

*AMD* age-related macular degeneration, *DME* diabetic macular edema, *ETDRS* Early Treatment Diabetic Retinopathy Study, *IRD* inherited retinal disease, *PDR* proliferative diabetic retinopathy, *PRP* panretinal photocoagulation, *VEGF* vascular endothelial growth factor

### Low-luminance visual acuity

Lighting conditions on charts play a greater role in the measurement of CS than VA due to the additivity of luminance and contrast effects [21]. Nevertheless, chart luminance still plays a crucial role when testing VA [22]. Therefore, target luminance on printed charts, projected charts, or digital screens is an important parameter that requires standardization when measuring VA [4]. Luminance is defined as the light emitted from a surface, with its intensity usually expressed in candelas per unit area of the emitting surface (cd/m^2^) [23, 24]. Target luminance refers to the level of light on the target display shown to a patient; to measure LLVA, luminance is decreased during testing, with either a filter placed between the chart and the eye tested or a digital screen with luminance control used. Specifically, LLVA is usually measured by placing a 2.0-log unit neutral-density filter (i.e., a filter that lowers luminance by 100 times, such as the KODAK WRATTEN Filter (Kodak, Rochester, NY, USA)) over the best correction for that eye and having the patient read the normally illuminated Early Treatment Diabetic Retinopathy Study chart. Thus, LLVA is a simple, inexpensive, and relatively rapid measure of visual function. LLVA has been recognized as a crucial factor when measuring VA. As VA and LLVA measure the same function under different luminance conditions, LLVA can be considered a more accurate surrogate for VA, which operates under optimal lighting conditions [4, 21]. Testing vision at decreased levels of luminance has been useful in the detection and monitoring of the progression of different stages of age-related macular degeneration (AMD), particularly geographic atrophy, compared with measuring VA alone [25], suggesting the utility of LLVA in the early identification of visual damage in patients with AMD. Other studies have demonstrated the value of LLVA in predicting the risk of future VA loss in patients with geographic atrophy due to non-neovascular AMD. In a cohort of 91 patients, LLVA was a strong predictor of the risk of losing VA in eyes with geographic atrophy, especially in patients with good vision at baseline [19, 26]. Although LLVA assessment is simple to implement and commonly used in the clinic, there is a lack of standardization in testing. Further investigation is needed to establish recommendations for target luminance levels. Wood et al. recommend recording the luminance threshold used for each LLVA score to improve consistency and reduce variability in the test results [27].

Another useful measure is the low-luminance deficit, which is calculated as the difference (in logMAR units) between LLVA and best-corrected VA measurements [19]. Pilotto et al. [28] demonstrated that LLVA and low-luminance deficit were significantly worse in patients with bilateral versus unilateral geographic atrophy. Furthermore, it was observed that low-luminance deficit is another potential predictive measure of subsequent VA loss and progression of geographic atrophy in patients with AMD [19, 29].

Tests based on low-luminance conditions have proved useful in the detection and prediction of geographic atrophy in patients with AMD. One possible explanation is that intact Müller cells are required for the normal functioning of prereceptorial visual pathways; the intact Müller cells preserve the original light beam that reaches any single Müller cell endfoot, and the light is then relayed to the cones [30]. The involvement of Müller cells has been observed in bilateral geographic atrophy secondary to AMD [28], and decreased sensitivity to light has been observed in patients with geographic atrophy even without changes in best-corrected VA [25]. Multiple studies have found Müller cell injury to be an important risk factor for progression in patients with atrophic AMD [31–33].

These data suggest that LLVA could be a complementary measure for evaluating visual function in patients with retinal diseases such as AMD, and it could also be employed as an alternative endpoint in future clinical trials. Although there is a lack of guidelines for applying LLVA in clinical practice, we recommend it be used regularly as a measure of visual function together with best-corrected VA.

### Contrast sensitivity

CS testing has been widely promoted as an important adjunct method to, or even a replacement for, VA testing. While VA measures the eye’s ability to resolve fine detail, it may not be able to adequately assess the ability to see large low-contrast objects such as faces [19]. For theoretical reasons, most investigators have used sine-wave grating stimuli: patterns consisting of alternating light and dark bars, which have a sinusoidal luminance profile. Sine-wave gratings vary in spatial frequency (bar width) and contrast [34], and this method determines the lowest contrast level at which a patient can differentiate optotypes from a background. CS can be explored either statically or dynamically [18], and a common clinically used tool is the Pelli–Robson CS chart [35]. Although the Pelli–Robson chart is widely used by clinicians, the Mars Letter Contrast Sensitivity Test is an alternative method with certain advantages. Dougherty et al. demonstrated that CS scores and repeatability were similar between the two methods but that making adjustments for contrast levels may enhance repeatability of the Mars test and make it an attractive alternative to the Pelli–Robson chart. Furthermore, the charts used for the Mars test are smaller and made of more durable materials, which may offer advantages for transport and use in different settings [36, 37]. Various techniques, some of which may be moderately time-consuming, have been adopted to measure CS, with many performed as a computerized test in which a display with a gray-level modulation is used. The advantages of computer-controlled acuity tests include more precise acuity measurements, increased efficiency, and greater reliability [38]. Most studies have generated significant findings in patients with early-stage retinal diseases without VA impairment [39], and several studies have shown CS abnormalities in patients with diabetes [40, 41]. CS has been reported to be a more sensitive measure of early retinal changes in patients with diabetes than VA [10], making it a useful tool for evaluating visual function in patients with diabetes with no visible ocular alteration. Other reports have also found CS to be a useful tool in the diagnosis, follow-up, and treatment of diabetic macular edema (DME) and diabetic retinopathy (DR) and even after panretinal photocoagulation treatment [42]. Furthermore, studies of macular function in patients with AMD have attempted to establish whether there is a relationship between CS findings and the initial stages of AMD, with promising results [18, 43, 44]. However, the results of a prospective design study by Owsley et al. [45], which investigated an association between CS and the incidence of AMD at 3 years of follow-up in eyes with normal macular health, did not show a CS deficit to be a predictable risk factor for the development of AMD. The report proposes that previous cross-sectional studies contained biases which could affect the reported results. Although the CS test needs to be standardized, we recommend it be adopted as a regular test in retinal clinical practice.

### Retinal fixation and microperimetry

Eye fixation is typically defined as the period that lies between two saccadic eye movements, while the patient is focused on a given target, in the absence of smooth pursuit eye movements [46]. In a 1996 report, Møller et al. [47] studied fixational eye movements as a measure for retinal diseases, and other, more recent reports have shown that eyes with AMD and DME can have alterations in retinal fixation tests [7, 48, 49]. Retinal fixation can be affected when there is damage to the fovea, resulting in a limited ability to focus on a single target or object. Instability in fixation is associated with a slower reading speed and reduced reading performance, affecting a patient’s ability to perform everyday tasks [47, 50, 51]. Patients experiencing vision loss due to foveal impairment frequently use a noncentral part of the retina for fixation, known as the preferred retinal locus [52–54]. When retinal fixation is measured through microperimetry, the unstable or noncentral fixation can also be quantified [52, 53, 55]. Fixation is continuously registered during a standard microperimetry test (dynamic fixation), performed to assess retinal threshold, but it may also be recorded as an isolated fixation task (static fixation) [56, 57]. Microperimetry automatically analyzes fixation stability through two different methods: the clinical classification method and the bivariate contour ellipse area analysis method [55, 56].

According to clinical classification, fixation is defined as stable if more than 75% of the fixation points are located within a 2° circle, centered on the gravitational center of all fixation points; relatively unstable if less than 75% of the fixation points are located within a 2° circle but more than 75% of the fixation points are located within a 4° circle; and unstable if less than 75% of all fixation points are located within a 4° circle [56]. This method does not allow for the typically elliptical distribution of fixation points and for the case of multiple preferred retinal loci. It also groups people with highly dissimilar fixation abilities into the same category; someone with good fixation could have 75% of fixation points within a 2° circle or 100% of fixation points inside a 0.5° circle [57]. The bivariate contour ellipse area analysis method has demonstrated good correlation with reading speed measures, suggesting that the quantification of retinal fixation parameters, primarily area, could also be used to quantify reading ability [57]. Although bivariate contour ellipse area analysis presents some limitations, it is the more desirable method to evaluate retinal fixation.

In patients with early AMD, Midena et al. [49] found that the use of microperimetry for the detection of decreased retinal sensitivity may be useful, and Al Shafaee et al. [58] found decreased retinal sensitivity in prediabetic patients when compared with normal controls when using microperimetry. In patients with neovascular AMD treated with intravitreal ranibizumab injections, Mathew et al. [59] investigated a correlation between the anatomical features of the macula with functional parameters such as location and stability of fixation, also measured with microperimetry. They found that VA, absence of subretinal thickening, intact subfoveal third hyperreflective band, and intact external limiting membrane were correlated with central and stable location of fixation, indicating a direct relationship between the integrity of the external retinal layers and central fixation. Similarly, disruptions in the ellipsoid zone band and retinal pigment epithelium were associated with reduced retinal sensitivity, despite VA being maintained [60]. Additionally, in patients with subtle vision loss due to AMD, microperimetry has demonstrated an ability to objectivize macular function, with certain advantages over measuring VA alone. This was demonstrated in the study by Tran and Herbort [7], where more than one-third of patients with AMD had a bad or very bad microperimetry performance in parallel with good VA, but patients still complained about their vision in daily situations.

Although time can be a limitation in microperimetry tests, current technology has improved the timing and quality of the examination, with evaluation schemes  taking no more than 5 minutes. Microperimetry and retinal fixation are therefore valuable tools for the assessment and monitoring of macular function in patients with AMD, DR, and DME. These two tests are potential indirect indicators of visual function, and we recommend their regular use in clinical practice. One of the advantages of microperimetry is the testing of the entire macular area rather than only foveal function.

### Reading performance

As many activities of daily living rely on reading, reading impairment is the most common complaint among patients with low vision participating in quality of life investigations [61, 62], with reading performance being a strong predictor of vision-related quality of life [13]. Measures such as reading speed are also reasonably different from near VA, with the latter being tested on a few sentences on a chart without considering the speed in which they are read. Reading longer parts of newspaper articles or books depends on a certain minimum reading speed. According to some studies, under 30 words per minute is insufficient for sustained reading [63].

Studies in patients with well-established AMD have reported a significant decrease in reading speed [64, 65], despite VA being within normal limits. This is because reading ability requires a larger intact retinal area [66]. Reading tests have also been found to be a useful parameter in evaluating the response to antiangiogenic treatment. In a prospective case series of 30 eyes with wet AMD, average reading speed increased from 59 words per minute at baseline to 85 words per minute after three intravitreal injections of anti-vascular endothelial growth factor therapy [63]. However, since the measurement of reading tests can be influenced by literacy level and cognitive factors as well as retinal fixation, its interpretation must be controlled for possible bias.

In order to better evaluate visual function, sentence-level reading acuity tests such as the Colenbrander, MNread, and Radner cards are preferred [67, 68]. Additionally, the International Reading Speed Texts (IResT; European Vision Institute, Brussels, Belgium) is a widely used clinical tool for assessing reading performance. Instead of single sentences, IResT uses standardized passages that are long enough to provide an accurate estimation of reading speed but short enough to prevent fatigue effects [68]. IResT has also been used to evaluate the effect of glare on reading in patients with AMD and glaucoma [69].

A study by Giacomelli et al. [70], which investigated the simultaneous association of several psychophysical measures with reading ability in patients with mild and moderate low vision due to AMD or DR, concluded that fixation instability and CS loss are the key factors limiting reading performance in these patients. As described previously, retinal fixation is highly correlated with both reading speed and reading performance. It has therefore been suggested that retinal fixation tests may encompass reading tests, and a unique retinal fixation test could be performed to also measure the reading ability of patients. However, reading performance is strongly linked to vision-related quality of life and its improvement is a high priority for patients threatened with loss of vision. Patient education and cooperation are critical for the efficient application of reading performance tests. We therefore recommend that regular assessment of reading performance be adopted in routine clinical practice, independent of assessments of retinal fixation.

## Measures of visual function requiring further optimization

The use of the following tests requires further research and optimization; some tools are time-consuming or are not standardized, and others require special equipment or conditions that are not always available (Table 1). Although we have reviewed these tests, we recommend caution until techniques have been more widely optimized and validated.

### Dark adaptation

Photoreceptors adapt to different levels of background light and ambient luminance through the bleaching and regeneration of visual pigments. Clinical dark adaptometry primarily measures the absolute thresholds of cone and rod sensitivity in complete darkness [71–73]. Although performing dark adaptation tests can take a long time, new instruments and strategies have been developed to make them more feasible in the clinic, by decreasing the duration of the tests but still maintaining their sensitivity [17, 74, 75]. However, standardization is still required.

A correlation between age and rate of rod sensitivity recovery during dark adaptation has been reported [76], and other studies have shown that when moving from bright light to lower illumination, vision can be decreased in patients with retinal diseases. In patients with AMD, rod adaptation [75, 77] and cone adaptation [71–73] are impaired, and dark adaptation has also been shown to be a highly reliable measure of early AMD across a range of measures such as rod intercept time, time constant of cone recovery, and rod-cone break [71, 74, 78]. Currently available evidence demonstrates that dark adaptation can be a potential biomarker for the diagnosis and progression of AMD.

For DR and DME, some initial studies have attempted to demonstrate the influence of metabolic fluctuation on alterations in dark adaptation (such as rod adaptation). This concept is based on energy consumption by depolarized rods under dark conditions [79, 80]. However, a randomized controlled trial which assessed 24-month outcomes of patients wearing an organic light-emitting sleep mask as an intervention to treat and prevent the progression of noncentral DME found that the mask did not confer a long-term therapeutic benefit on non-center-involving DME due to the dynamic nature of the disease [81]. Nevertheless, dark adaptation has been shown to be useful in the early detection and prevention of retinal damage caused by diabetes mellitus [80]. In DR, Hsiao et al. [82] discovered a correlation between optical coherence tomography angiography and the rod intercept on dark adaptation. Decreased deep retinal vascular perfusion density and impaired dark adaptation response were also observed as DR severity progressed.

The use of dark adaptation as a clinical outcome measure or practical diagnostic tool in retinal diseases is hampered by a long test duration, high participant burden, limited cooperation from patients (with complaints such as visual fatigue), a lack of standardized dark adaptometers, and a lack of reproducibility. Further research is needed to make dark adaptation ideal for regular use in clinical practice.

### Binocular vision testing

Visual function is typically evaluated monocularly. There is strong psychophysical evidence that visual performance is better under binocular than monocular observation (known as binocular summation), with improvements in high-contrast VA and CS being 10% and more than 60–70%, respectively [83, 84]. However, in patients with AMD in whom one eye is affected more than the other, or in whom monocularly preferred retinal fixation points are not in corresponding positions, VA in the better-seeing eye can be affected by the worse eye when the patient is assessed under binocular viewing conditions [85, 86]. Conversely, other studies have shown that fixational ocular motor control and VA are different depending on whether tasks are monocular or binocular, demonstrating that the performance of the worse-seeing eye can improve under binocular tasks [16, 87]. Binocularity is an additional measure of VA; it provides a more realistic measure of a patient’s functional visual performance, and after further research and standardization, this test could be performed daily to capture real-life situations of visual function.

### Color vision testing

Several studies have investigated the relationship between color vision abnormalities and retinal diseases such as DR and DME. Bresnick et al. [88] used a Farnsworth–Munsell 100-hue test to explore a direct link between the severity of DR and DME and color discrimination. They found that the magnitude of a blue-yellow discrimination defect correlated significantly with the severity of overall DR and the severity of macular edema and hard exudate formation, thus supporting the use of color discrimination tests together with VA measurements for the management of DR and DME [88, 89]. However, Farnsworth–Munsell is time-consuming, so tests such as the Cambridge Colour Test or other computer-based tests can be used to overcome this limitation [90–92]. A more recent study established that VA does not always correlate well with clinical severity in DME [93], while other reports have demonstrated that patients with DME are three times more likely to have impaired color vision than patients with DR alone [89, 94].

Color discrimination tests enable a better understanding of treatment effect in patients with DME. In a study by Abdel-Hay et al. [8], both red–green and yellow–blue chromatic sensitivity were assessed in patients with DME treated with intravitreal dexamethasone. The results showed that red–green chromatic sensitivity can be a useful biomarker in monitoring treatment efficacy in DME, in addition to VA and central sub-field retinal thickness.

Color discrimination tests could also be useful for monitoring patients with advanced AMD. Dorrepaal and Markowitz [95] found that patients with late AMD and poor VA can discern smaller targets on a red-on-yellow color scheme than on achromatic white-on-black charts.

In summary, several studies have shown that color discrimination can be a useful additional tool in the early stages of DME and also for intermediate to advanced stages of AMD and DR [8, 94, 95]. Although promising results have been observed, many of these are from initial studies with low statistical weight and basic design, so further studies are required to determine whether color discrimination tests should be established in regular clinical use.

### Visual recognition tests

Most patients with advanced AMD develop a central scotoma due to atrophy of the macula, where the density of photoreceptors is extremely high [12]. This affects higher-level visual functions such as reading and face recognition [96, 97]. Some studies have therefore explored visual recognition of objects and scenes in patients with AMD, with results showing that these types of tests are an additional simple and reliable tool to determine the severity of AMD and impact on patients’ daily activities [12]. Studies exploring the utility of visual recognition tests in relation to geographic atrophy as a biomarker of progression would also be of interest. However, although promising, this type of test is new, with limitations due to the patient cognition level and also its lack of standardization. Further studies are needed before its use in routine clinical practice.

### Shape discrimination

Metamorphopsia, aniseikonia, and other shape alterations are common symptoms of visual function disturbance in various macular disorders and can often be disabling for the patient. Despite the prevalence of these symptoms in common retinal diseases such as AMD, there are no clinically validated tests [11, 98–100]. Several tests, including preferential hyperacuity perimetry [101] and MonCV3 (Metrovision, Pérenchies, France), are currently undergoing investigation and therefore remain a key area of further research and evaluation before such tests can be used routinely. Some difficulties associated with testing for shape discrimination include the lack of standardization and considerations regarding a patient’s cognitive level. Additional studies are also needed to determine the place of this technique in clinical practice or for patients to self-monitor AMD.

## Conclusions and further considerations

The slow progression of some retinal diseases can present challenges in clinical trials, as currently used endpoints of acuity are relatively insensitive to early disease progression [75]. In the same way, an unmet need remains to differentiate the long-term effect of intravitreal drugs, mainly in neovascular AMD where best-corrected VA seems unchanged. New and additional functional endpoints are required to fully understand the early stages of macular disease, its progression, and the response to treatment.

While this review largely focused on AMD and DME, these techniques are also applicable to other retinal diseases. LLVA, CS, retinal fixation, and color vision testing are promising tests for inherited retinal diseases [102–104]. In addition, binocular vision testing could be useful in assessing inherited retinal diseases and nystagmus [105]. Other methods for visual field testing, such as multifocal electroretinograms and multifocal visual evoked potentials [106–109], can be valuable in the differential diagnoses of retinal and optic nerve diseases [106, 107] and can assess visual field effects not yet present on automated perimetry [110]. While electrophysiologic tests are more objective measures of visual function than psychophysical tests, the multifocal visual evoked potential method requires specialized software to analyze results and is not applied in most routine clinical practices [107]. Frequency-doubling technology perimetry can be applied as an alternative exploratory method to detect loss of visual field [111]. Although frequency-doubling technology methods are used in macular assessment, they are mainly applied in the assessment of the mid-peripheral/perimacular region to identify visual field defects in optic nerve-related diseases [112].

Additional tests to measure and quantify other aspects of visual function have shown promising results in independent studies [48, 49, 113], and some tests are closer to being used in clinical practice (CS, retinal fixation, LLVA, and reading test/ability). Some are very useful for application in all retinal diseases, in that they are able to evaluate several functional parameters, are less time-consuming to perform, and are highly reproducible. Ultimately, many of the tests discussed can help better characterize the visual function affecting a patient’s quality of life.
